# Need Satisfaction Indirectly Associated With Lower Caregiver Burden & Cytokine Production in Dementia Caregivers

**DOI:** 10.1093/geroni/igaf122.3628

**Published:** 2025-12-31

**Authors:** Kate Bishop, Jensine Paoletti-Hatcher, Daniel L Argueta, Kelly N Brice, Bryan T Denny, Charles Green, Samantha Henry, Christopher P Fagundes

**Affiliations:** Portland State University, Portland, Oregon, United States; Portland State University, Portland, Oregon, United States; Rice University, Houston, Texas, United States; Rice University, Houston, Texas, United States; Rice University, Houston, Texas, United States; UTHealth, Houston, Texas, United States; Baylor College of Medicine, Houston, Texas, United States; Rice University, Houston, Texas, United States

## Abstract

Spousal dementia caregiving is a chronic stressor linked to systemic inflammation and poor health. Self-determination theory (SDT) may provide a useful framework for identifying which caregivers are at the greatest risk. SDT posits that psychological needs satisfaction (PNS) may increase the relative autonomy caregivers perceive within their relationship, contributing to increased well-being overall. We tested a causal association hypothesized in SDT: higher PNS should be indirectly associated with greater well-being through more autonomous motivation. We examined the indirect effect of PNS on self-reported caregiver burden and cytokine production through autonomous motivation for caregiving in cross-sectional analyses of 108 caregivers (M = 72.18, SD = 7.69; 72.22% women; 53.70% advanced degree holders). Participants reported demographics and health information, caregiver burden (ZBI), autonomous motivation for caregiving (modified Self-Regulation Questionnaire; α = .84), and relationship-oriented PNS (modified Needs Satisfaction Scale; α = .86). LPS-stimulated cytokines (IL-6, IL-10, TNF-α, and IL-1β) from whole blood were combined in a linear composite (α = .85) before we ran mediation analyses in lavaan. We found that higher PNS was associated with lower cytokine production through higher relative autonomy (indirect effect = ˗0.15, 95% CI [˗0.28, ˗0.013]). We also found that higher PNS was associated with lower caregiver burden through higher relative autonomy (indirect effect = ˗0.11, 95% CI [˗0.21, ˗0.023]). Dementia is degenerative and requires caregivers to adapt regularly to meet changing demands; longitudinal or time-lagged research is needed to capture the role of PNS as a possible causal factor in caregiver resilience.

